# Experiences and coping strategies of women caring for their husbands with cancer at the Cancer Diseases Hospital in Lusaka, Zambia: a descriptive phenomenological approach

**DOI:** 10.3332/ecancer.2023.1572

**Published:** 2023-07-13

**Authors:** Patience Mbozi, Patricia Katowa Mukwato, Victoria Mwiinga Kalusopa, Christopher Simoonga

**Affiliations:** 1 Department of Nursing, Faculty of Health Sciences, Chreso University, Lusaka 10101, Zambia; 2 Department of Basic and Clinical Sciences, School of Nursing Sciences, University of Zambia, Lusaka 10101, Zambia; 3 Chreso University, Lusaka 10101, Zambia

**Keywords:** cancer, wives of men with cancer, spouse, caregivers, coping strategies, coping, experiences

## Abstract

The Cancer Diseases Hospital (CDH) 2019 annual report revealed an upsurge in the number of new cancer patients accessing services from 35 patients in 2006 to 3,008 in 2019. This study explored the experiences and coping strategies of women caring for their husbands with cancer attending the CDH. A phenomenological research design was used with stratified purposeful sampling. Data were collected using an interview schedule and analysed using thematic analysis. The women’s challenges included mobility difficulties and hospital admissions/problems; socio-economic problems, psychological and emotional distress; and caregiving liability and spiritual anguish. The benefits that female spouses experienced during caring for their loved ones included knowledge about cancer and infection prevention, a strong marital relationship, tolerance and perseverance, resilience and hope and good relationship with other caregivers. The women’s needs included financial support, physical needs, psychosocial counselling, caregiver accommodation, time off from caregiving, information needs and sexual intimacy and contact. Their coping strategies included spiritual support from spiritual carers, prayer and meditation, music and storytelling, social support and a good marital relationship. The findings demonstrate that wives of patients with cancer experience many challenges in their caring journey. Nurses must anticipate and/or intervene as part of their nursing practice to reduce the negative impact on female caretakers in this situation. Hospital standard operating procedures must be developed to put both the patients and their caregivers at the centre of oncology nursing care, particularly in settings with limited allied professional support, e.g., psychologists. Caretaker coping strategies highlighted in this study must be made available for both the patients and their wives, e.g., linking wives to trained spiritual carers upon their husband’s admission to the hospital, to aid a smooth caregiving experience.

## Background

Cancer is a global health problem with high morbidity and mortality rates [1]. The Cancer Diseases Hospital (CDH) in Lusaka, Zambia, has seen a rise in the number of male patients with cancer from 581 in 2015 to 1,126 in 2019 and these patients are mostly cared for by their wives [2]. Milimo *et al* [3] reported that women in Zambia are predominantly involved in performing household chores and participate in income-generating activities to help support their families. Evans [4] agrees that women in Zambia are generally categorised as housewives, unlike men who are seen as masters of their own households. Geisler [5] further asserts that despite equal rights guaranteed in the Constitution, women in Zambia are seen to be below their husbands’ authority and cannot do anything without their husbands’ permission. Reigada *et al* [6] state that caregivers are the greatest support of patients in a patient diagnosed with cancer or any other incurable illness. In Zambia, the average family has five children [7], but can even go up to ten children.

Walubita *et al* [8] highlighted in their study that women in Zambia perform multiple tasks including caregiving of their loved ones in the hospitals and face social stigmatisation for caring for a patient with cancer. They further asserted that caregivers of patients with cancer face inadequate finances for health care services and lack knowledge about cancer. These findings align with research on female caregivers of men with cancer from other countries. Sercekus *et al* [9] found that in Türkiye, female spouses are the ones who are most affected during the care of their loved ones. These women experience challenges such as stress, fear, anorexia, hypertension, anxiety and weakened immune system. Tolbert *et al* [10] stated that spouses of men with cancer in the US reported limited social activities because of the gradual change in their roles both in the community and at home, such as from being wives to breadwinners. Takeuchi *et al* [11] also found that women in Japan reported having insufficient time to take care of their children and watch movies or sports when caring for their spouse with cancer. Other women in Japan and Türkiye have been documented to have had to quit their jobs and many lost their jobs as they spent more time taking care of their loved ones who were admitted to the hospital [9, 11]. Tolbert *et al* [10] argue that women actually felt unsupported by their husbands despite giving them moral support and care and quitting their jobs. According to a study done in The Netherlands on caregiving experiences of partners of patients (men and women) with colon or rectal cancer, Nijboer *et al* [12] added that the caregivers reported being deprived of their social activities and suffering from stress, anxiety and psychological distress.

### Caregiver coping

Walubita *et al* [8] reported that the caregivers of paediatric patients with cancer in Zambia depend solely on spirituality and traditional healing for their patients. Sibulwa *et al* [13] found that both adolescents with cancer and their caregivers in Zambia used spirituality (prayer) a coping mechanism. LeSeure and Chongkham-Ang [14] asserted that findings from their systematic review revealed a sense of accomplishment felt by caregivers when they see that their role of caring for their loved ones and making them comfortable is recognised and reinforced. Boatemaa Benson *et al* [15] reported that caregivers of patients with breast cancer in Ghana employed several strategies to enable them to cope during the care of their loved one in the hospital and at home such as praying, performing household chores, watching movies, sowing, tailoring, carrying out handcrafts and using faith. Semenova and Stadtlander [16] recommended rendering counselling, support and information to the spouses of patients with cancer in the US. The authors also reported some positive spousal experiences of caregiving such as their motivation to care and give moral support.

Caring for patients with cancer across the world has been documented as stressful and with negative impacts. This study aimed to explore the experiences and coping strategies of women caring for their husbands with cancer attending the CDH in Lusaka, Zambia.

## Methods

### Study design, setting and participants

This exploratory qualitative study [17] used a descriptive phenomenological approach [18]. The study was conducted at the CDH in Lusaka Province, Zambia, from October 2021 to January 2022. A stratified purposeful sampling method was used. The sampling frame was first divided into strata according to categories of cancer diagnosis to have a general view of the caregiver experiences, and then a purposeful sample was selected from each stratum [19]. This sampling design facilitated group comparisons.

### Study population

The study population comprised the female spouses caring for their husbands who have cancer attending the CDH in Lusaka; however, the type of cancer in men was not prespecified because the researcher sought to explore the general experiences of female spouses caring for their husbands with cancer. To compare the women’s needs, the experiences and coping strategies of spouses of men with various kinds of cancer were included in the study. Eligibility criteria included women 20 years or older who were currently caring for their husbands with cancer attending the CDH, whether the patient was an outpatient or inpatient. Women who cared for their husbands with cancer who were now cancer survivors but still attending the CDH (outpatient or inpatient) and women who had husbands with cancer who had died were also eligible for the study. Exclusion criteria included women who were nursing another relative apart from their husband with cancer, women who were nursing very ill husbands with cancer, women who were nursing husbands at the end of life and male caregivers of women with cancer.

### Methodology

Two field research assistants, who were trained by the researcher for 3 days, reached out to participants at CDH to conduct in-depth interviews. An interview schedule (designed by the researcher to address the research objectives) was used to collect data in face-to-face interviews. All interviews were conducted in English. The setting for the interviews was a confidential room at CDH. Interviews were recorded using a tape recorder and transcribed word by word by the researcher [18]. The semi-structured interview schedule was intended to generate rich data on the women’s experiences and coping strategies [20]. The field research assistants probed for further information during the interviews to ensure that participants understood the questions asked [21].

Field notes were collected and maintained throughout the data collection process. The transcriptions were compared by the researcher with the information in the field notes to check and identify errors made on the transcriptions [17].

### Trustworthiness of the data

Lincoln and Guba [48] suggest four dimensional criteria for study rigor, credibility, dependability, confirmability and transferability [49]. Confidence in the truth of the findings denotes credibility; applicability to other settings or contexts denotes transferability; consistency of the findings denotes dependability and the degree to which the findings are shaped by participants and not researchers denotes neutrality [48, 49].

#### Credibility

The women in this study participated voluntarily and were told that they could withdraw whenever they wished. During interviews, probes and prompts were utilised to obtain detailed data using the interview guide. Participant answers were paraphrased to ensure that the recorded data was what the women had stated. This facilitated checks and confirmations during the interviews that the participants’ words were accurately recorded. Interviews were audio recorded and transcribed. Emerged themes were verified at the end of data analysis, and the participants were recontacted and asked to confirm if the themes were a true reflection of their responses. The researcher’s feelings, perceptions and thoughts were suspended so as not to interfere with the participants’ views and experiences [17, 18, 22].

#### Dependability

The research design and the risks and benefits of the study were explained and given in writing to the participants so that dependable results would be produced. Dependability was ensured by the use of recording devices, detailed transcripts, field notes, audit trials and regular consultations with oncology nurse colleagues and co-authors during analysis and interpretation. Reflexivity was achieved by ensuring that each of the three data collectors recorded their observations and feelings while conducting the interviews and these were discussed every other day.

#### Confirmability

To ensure confirmability, the findings of this study reflected the participants’ voice and not the researcher [18]. A description of the study methodology, which allowed for scrutiny of research findings, was given to the participants [17, 18]. Supervisors and oncology nursing colleagues reviewed the selected transcripts and themes.

#### Transferability

To ensure transferability, this study described here clearly highlights the background, data collection methods, sampling method, sample size and study limitations. The research design and sampling method have been explained extensively in this section [17, 18, 22].

### Ethical considerations

The University of Zambia Biomedical Research Ethics Committee (REF. 1918-2021) and National Health Research Authority gave authorisation to conduct the research. The study site (CDH) also granted permission. Informed consent was obtained from participants after a patient information sheet describing the purpose, benefits and risks of the study was thoroughly explained to them. Participants were assured of anonymity that they were allowed to withdraw from the study without consequence if they wanted to, and participation was voluntary.

### Statistical analysis

Thematic analysis was conducted by searching across the data set to identify, analyse and report repeated patterns [23]. During data analysis, the researcher read and reread the transcripts several times to understand the content of what the women had said. This enabled the researcher to find meaning units or content topics shared by the women; these were coded into categories through a coding framework and the content in each of the categories was analysed, and themes were identified, reviewed and defined by both the researcher and her supervisor, a nurse faculty member with expertise in qualitative research. When each participant’s transcription was received, data analysis commenced, and additional participants were enrolled in the study until no new information was identified [24]. Repetitive were checked and these were gathered together. The examination of the transcriptions was conducted several times to determine additional emergent themes [18, 21]. The researcher shared the codes and the identified themes from the analysis process together with the transcriptions with her supervisor for verification and with the researcher’s colleague for peer review.

## Results

**Figure 1.**
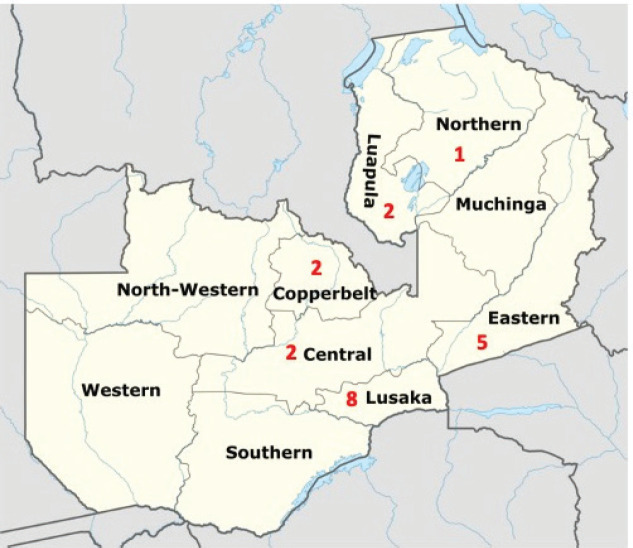
Home locations of the study participants (N-number) by Zambian Province (https://commons.wikimedia.org/wiki/File:Zambia_provinces_named.png).

The total sample was 20 women. Seven were 20–29, seven were 40–49, three were 30–39 and three were 50–69 years old. All the women were married, and 2 had no formal education, 11 had attained primary education followed by 5 with a secondary education and 2 had a tertiary education. Twelve women were self-employed, three were civil servants and five were unemployed. Six women were Pentecostal, three Roman Catholic, two Jehovah’s Witnesses, two New Apostolic Church, two Reformed Church, two Seventh Day Adventist and one each Baptist, Methodist Church and United Church of Zambia. Eight women reported less than Zambian Kwacha 1,000 (~USD 50) income per month, six K1,000–K3,000, four K3,000–K5,000 and two above K5,000 income per month. Eight women came from Lusaka, five from the Eastern Province, two each from Central, Copperbelt and Luapula Provinces and one from the Northern Province (Figure 1). Table 1 presents the demographic characteristics of the participants and Table 2 presents the cancer diagnoses and treatments of the women’s husbands.

### Experiences of women caring for husbands with cancer

Themes that emerged from the data were not predetermined. The themes were broadly classified as challenges/barriers, facilitators/benefits, needs and coping strategies. The women’s challenges included mobility difficulties and frequent hospital admissions, inadequate caregiver accommodation and poor hospital environment, socioeconomic problems, psychological and emotional distress, caregiving liability and childcare, spiritual anguish and some participants stated that they learnt nothing. Facilitators or benefits of caregiving for spouses of men with cancer included knowledge about cancer and infection prevention, a strong marital relationship and development of good virtues such as tolerance, perseverance, resilience and hope and good relationships with other caregivers and healthcare workers. Coping strategies included spiritual support from spiritual carers, prayer and meditation, music and storytelling, social support, ignoring and avoiding distressing situations and good marital relationship. The women’s needs that emerged from the data included financial support, physical needs, psychosocial counselling, sexual intimacy and contact, caregiver accommodation, time off from caregiving and information need. Table 3 presents the themes identified from interview data.

### Challenges/barriers

There are many challenges that female spousal caregivers go through when caring for husbands with cancer in Zambia. The challenges and barriers identified from the qualitative analysis are highlighted below.

#### Mobility difficulties and hospital admissions

*Frequent hospital admissions.* Many women highlighted that they experienced frequent hospital admissions due to their husbands’ cancer. Most expressed that they accompanied their husband to the hospital for his treatment such as chemotherapy, on average for 3–7 days. Some participants said that they stopped the usual schedules of their lives due to frequent admissions.

*‘Uhm….like when, like when we start chemotherapy, they admit us for 3 days. So, we find that what to buy becomes a lot for us to meet the three days or… he needs a lot of things maybe this week you will manage but the other week you won’t’. Respondent 1.*

*‘He’s in hospital [and I] am always there I can’t go to work am always there by his bedside am not able to go to church cause he will be there from Thursday sometimes till Saturday’*. Respondent 6.

Some participants expressed that they were not even able to adequately take care of their children at home because of their husband’s frequent admissions.

*‘Uh, it is a challenge to look after our children causing to the fact that we are ever going to the hospital for his treatment’. Respondent 5.*

*‘The challenges that aaah...it’s now four months we are away from home yes’.* Respondent 7.

Some women stated that hospital admissions took most of their time.

*‘Most of the times you find that like this we are in hospital uhmmm…’. Respondent 15.*

Others expressed that they feared that their husbands would be admitted when they came to CDH even for follow-up services and review of their patient’s cancer condition.

*‘I would ask him to go to the hospital; he would refuse because he was scared. I would ask ‘you are scared of what?’ He would say he’s scared of been admitted’. Respondent 9.*

#### Transportation difficulties

All the participants expressed that they had transportation difficulties whilst caring for their husbands. They mentioned that their transportation challenges included the distance they had to travel before they could come to CDH to access the cancer services and the frequency of coming to and from CDH during their husbands’ illness.

*‘Well, we have challenges with transport, ummmm…to move to and from home to the hospital is a challenge at times’. Respondent 5.*

*‘Challenges like you know loss of finances, and then Ummm... Moving is hard, it’s Ummm mobility... Coming there to Lusaka every time when he’s review is due...’ Respondent 6.*

*‘It’s transport, because here we come three times...yes, so this 3^rd^ time, we thought no, this want is getting worse, the neck will be cut...’ Respondent 11.*

#### Inadequate caregiver accommodation and poor hospital environment

*Inadequate caregiver accommodation.* The women reported that they struggled finding where to sleep at night or when their husbands were sleeping or resting at any time of the day.

*‘Because am here I have nowhere to sleep, I just sleep down, the challenges are a lot, all I want is for my partner to get well because life here is hard’. Respondent 3*

*‘When we are admitted to the hospital, he will be sleeping, I just go to sit on the chair, I do not have where to sleep’. Respondent 2*

The women also complained of being in a poor hospital environment, which was a challenge. They talked of an inadequate water supply, dirty water on the hospital floor and dirty toilets and bathrooms.

*‘Coming there to Lusaka every time when his review is due...Mmmm... Challenges are there at the hospital, there is no water, yeah... The bathrooms maybe there are dirty, my husband is that person who doesn’t like dirty, so it is very difficult to go to the toilet when he finds water on the floor’. Respondent 6*

#### Socio-economic problems

*Disrupted social life.* Caregivers reported having a serious disruption in their social lives including interactions with family, social gatherings, friends, work, jobs and entertainment. They stated that they could not visit their families and friends or attend social gatherings such as funerals and churches. They felt that they were isolated from the world due to the situation of caring for their spouses with cancer.

*‘There is no entertainment, I’m being isolated, I can’t visit my friends no see them and family as I’m looking after him all the time’ Respondent 4.*

*‘I can’t socialize with my friends, I can’t visit family, I don’t normally go to church, on social, I can say am not doing anything’. Respondent 6*

*‘I can’t tell any entertainment sprees like I used to, I live in isolation in taking care of him uhmmm... I don’t visit my friends and family like I used to before. Finances are also affected as I don’t go for work and he doesn’t too so income is low nowadays’. Respondent 10.*

*Financial constraints.* Financial constraints came out loudly in the analysis. Many women expressed that they lacked the money to meet their needs and their patients’ needs. This could also be attributed to the fact that participants left their homes to live in the hospital. Others mentioned that the lack of money was a result of frequent hospital admissions, long duration of hospital stays and inability to find money due to their caregiving role.

*‘So we find that what to buy becomes a lot for us to meet the three days or… he needs a lot of things maybe this week you will manage but the other week you won’t manage and we need to stay in for three days’. Respondent 1*

*‘I worry about what they will eat because at least when am home,.….a lot of bills at home, I have to buy water and pay for electricity, just a lot……..’ Respondent 2.*

*‘Well, we have challenges ummmm…to move to and from home to the hospital is a challenge at times, finances have become a challenge, ummm….’ Respondent 5.*

Some women highlighted that a lack of money led to them being subjected to the hospital food that they had to eat, even on days that they did not even want such type of food, all because they did not have control (with money) over what they should eat.

*‘The other challenge is finances which we currently are lacking now, it’s hard to buy the food he wants/needs’. Respondent 10*

*‘Yes even the food in the hospital is not enough, no proper diet, if you don’t have money, so sometimes if you don’t have money they just bring the food you don’t like’. Respondent 15*

Other participants highlighted that despite being in hospital, they also needed to run their homes by continuous payment of household bills such as rent, electricity and water.

*‘Since I’m here taking care of him, there the house is not being paid for, at least when I was outside I could give my in-law to work some and I pay for the house. But now money for rent is now hard to find so I was thinking of going to remove my things and take them to my mother, and try to pay the arrears for 2 months then we see how far the sickness is going’*. Respondent 14

*Again, rentals again food prices have gone up and we do not do anything, so it’s hard. Respondent 1*

*Health care bills.* The women reported that they had challenges with payments of health care bills. These are health care bills that are not normally funded by the Government of the Republic of Zambia (GRZ) through the Ministry of Health such as laboratory and diagnostic investigations, hospital bed space payments (for those in high-cost wards), foodstuffs other than what the hospital provides and medicine that has run out of the hospital pharmacy.

*‘Lifestyle (the way we stay) at home and the medicine what is it ‘Mesina’ is a problem, so now it has given us a problem, to buy. Even the way we stay at home it’s slow we not able to buy food’ Respondent 13.*

*‘Some of the challenges I forgot to say, are the drugs are too expensive sometimes we run out, we don’t have money’. Respondent 6*

*‘My question is on drugs, because the pharmacy most of the time they don’t have drugs, you know drugs are supposed to be there’. Respondent 15*

*Inadequate hospital food supply.* The women highlighted that they experienced inadequate foodstuffs during their caregiving role. Some mentioned that the hospital food was not sufficient to cater for the patient and them. While others added that the free hospital food that they were given was not according to their diet preferences, as the food served is generally the same for everyone. Moreover, women stated that they lacked food due to long hospital admissions and generally, lack of funds.

*‘Money is a problem for food, for example, when some have been discharged, they leave food for me sometimes money. Like today I was given a k50 buy food from a well-wisher’ Respondent 9.*

*‘The other challenge is finances which we currently are lacking now, it’s hard to buy the food he wants/needs’. Respondent 10*

*‘And all what else... Yes even the food in the hospital is not enough, no proper diet, if you don’t have money’ Respondent 6.*

*Career halt.* Many women reported having had a career halt since they were self-employed or civil servants. Some were retired and others were unemployed. The self-employed women reported having a business that they were running, and others were farmers. Many women said that everything else stopped, as they focused on caring for their sick husbands.

*‘Everything has come to a standstill because of my husband’s condition’. Respondent 1*

*‘The challenges that aaah...it’s now four months we are away from home yes. Am not able to take care of the children we are away from them and the family. And...As of now as I was working doing part-time work as a teacher, I have held on and also his lying on the bed his not working, those are the challenges am facing’. Respondent 7.*

*‘It has a great impact on my occupation as I do not report for work due to my husband’s condition’*. *Respondent 10.*

*‘Concerning my work, I stopped because the other week I’m in Lusaka the other week I’m in Copperbelt’. Respondent 6*

*Inability to continue their businesses.* The women also mentioned the disruption of their businesses. These self-employed participants relied on their businesses for their upkeep. These are small-scale businesses that participants ventured in to take care of their families such as small-scale farming, a market stall, restaurant work or selling second-hand clothing,

*‘Because, me I do business and he also does business so, business it’s like it’s at a standstill, because we need to take the children to school we have stayed for a long time meaning we won’t be able to take our children to school’. Respondent 1*

*‘My business is down even the capital we ate it, a lot of bills at home, I have to buy water and pay for electricity, just a lot……..Things like my business, I can no longer do that, even back home. I can’t live the house, and leave him alone’ Respondent 2.*

#### Role and lifestyle changes

The women expressed that their husbands were fundamentally breadwinners in their households; therefore, their sicknesses led to role and lifestyle changes. They reported that this role change happened in that wives became breadwinners by ensuring that house rental and house bills were paid, and food was readily available in their homes and in the hospital for their husbands who were patients. They further reported that lifestyle changes happened when their life completely changed from the usual one to a life where they would live and care for their sick loved ones for a long time.

*‘Uhmm...... When you have someone who was an active breadwinner, doing the jobs but now he can’t because he is sick and now...it’s remained to me who is managing for the two of us yes...’ Respondent 7.*

*‘No... I have learnt, because that one is my helper, now he’s sick, you you are there, so who is going to help, so who is going to help me I can’t help, a man helps isn’t it?... Where he goes, he brings something now, like this is nothing no... It’s hard I have learnt it’s hard when your friend is sick it’s hard…’ Respondent 11.*

*‘Life has become hard because it’s different from what it was before and now, because now you have to wait it’s like you asking from someone and for them to help you, you find maybe they have their own problem so they can’t manage that’s the biggest problem I’ve found’. Respondent 15*

#### Psychological and emotional distress

*Psychological distress.* The women expressed many attributes of psychological distress. They reported feelings of worry, anxiety, frustration and fear while caring for their husbands. They stated that they would worry about the patient’s illness, and what he would eat. Notably, the women did not express anger or resentment towards their husbands, but did address their husbands’ anger. For example, some women mentioned that they were worried about how they would respond to their husbands’ changed moods such as anger – obviously due to the disease process. Some women expressed that their worry was about their household dependents including children, of how they would survive without them since they were in the hospital.

*‘Uhmmm… the impact is huge, especially mentally it’s affected me and psychologically because sometime you find that am not thinking straight, am thinking about his illness’. Respondent 1*

*‘It has brought psychological torture to me, but you know for his sake and try to stay calm’ Respondent 4.*

Some women described their fear as in the way their husband would treat them and mentioned that their husbands had anger issues and would normally take it out on them as wives. Others reported that when their husbands complained of pain, they felt pain.

*‘The challenges are many... But mostly it is anger issues; my husband gets to get angry whenever he’s in pain and takes it out on me’. Respondent 10*

*‘It has brought an impact because you know what, when they tell you that you have this disease eeeh…..fear comes in your heart; what is this disease now?’ Respondent 7.*

*‘I have children I’m looking after and get worried of how they are surviving without us. I wonder if they have eaten and the like’. Respondent 9*

*‘It has greatly and really affected the psychologically to the point that at times I really concentrate on what I’m supposed to do’. Respondent 15*

*Loss of sexual interest.* The women reported a loss of interest in meeting their husbands sexually due to their husbands’ condition. Wives of men with genitourinary and gastrointestinal (GIT) cancers reported having reduced their sexual desire.

*‘As I’m here nursing my husband…..This makes my mind go astray yeah. Hahahaha I don’t have any sexual needs at the moment I’m not even thinking about it’. Respondent 7*

*‘I stopped everything, I can’t go anywhere I can’t do anything, even the feeling of wanting to be with a man it has passed I don’t have at this time, and I just want to see the patient’. Respondent 15*

*‘Because you know what uhhh...….why …..I’m hahahaha I don’t have any sexual needs at the moment I’m not even thinking about it’. Respondent 7*

#### Caregiving liability and childcare

*Inability to provide child support.* The women stated that they were not able to meet their children’s support due to their caregiving role. They reported that apart from their caregiving role, they also had families to take care of, especially children. They reported worrying about their children’s wellbeing during their absenteeism from home, payment of school fees, and food and baby care (babysitting). GRZ provides free education from grade 1 to 12, but parents or guardians have to pay for uniforms, books and other school supplies.

*‘The challenges that aaah...it’s now four months we are away from home yes. Am not able to take care of the children we are away from them the family’. Respondent 7*

*‘I took the children to my mother I have left them in the hands of my family, because that house we left it locked that’s why I’m thinking of going to remove the things, pay the arrears then I can be free of thinking about the house’. Respondent 15*

*‘I have children I’m looking after and get worried of how they are surviving without us. I wonder if they have eaten and the like’. Respondent 9*

*Caregiving burden.* The women reported that they had a caregiving burden while caring for their husbands. This caregiving burden included taking care of their husbands’ needs whilst in the hospital such as bathing, feeding, looking for food for him, offering psychological and emotional support, and managing the husband’s side effects. Participants also talked about having a lack of accommodation where they could sleep while caring for their loved ones. Caregivers complained that they had no proper place to sleep as they just lay down on the hospital floor, which is quite burdensome for caregivers whose patients have been admitted for a long time.

*‘Yes am at the bed side and when he is in hospital am always there I can’t go for work am always there by his bedside am not able to go to church cause he will be there from Thursday sometimes till Saturday, then when he is out of the hospital he becomes very weak with the drugs so I can’t leave him alone at home’. Respondent 6*

*‘We are passing through a lot I tell you... kikikiki (laughing) it’s only God who knows... this wound on the neck is getting worse, the neck will be cut... Because most of the time it’s me who washes it, cleans it, and tying it, and I feel bad when doing that...’ Respondent 11.*

*‘The experience is okay, because when I look back, he also used to take care of me when he was working so now that his sick, they is nothing I can do but take care of him, am the only one that can do that because he’s not able to do that on his own’ Respondent 15.*

#### Spiritual anguish

*Spiritual distress.* Some women expressed spiritual distress that could not be ignored as a result of what their husbands were going through. They reported that they could not attend church services or meet their spiritual carers (clergy) due to their caregiving role. Some expressed that because of their absence from church services, they became spiritually distressed.

*‘Yeah Mmmm.... It’s hard I tell you, now I say God this condition is becoming too much’ Respondent 11.*

*‘Spiritually I’m affected because Ahhhh... I don’t attend church like I used to’. Respondent 10*

*‘We are passing through a lot I tell you... kikikiki (laughing) it’s only God who knows...’ Respondent 15.*

*‘Yes yes yes most of the time when I feel astray us, as New Apostolic church members we have leaders in our church, I call my leader uhmmm...’ Respondent 7.*

*‘Praying I don’t pray, I just pray when I’m sitted here we don’t come out to go to church’. Respondent 9.*

#### New perspectives about life occurrences

The women explained that caring for their husbands with cancer brought about a new perspective on life. Others said that initially they thought their husbands had problems other than cancer, until the diagnosis was confirmed, which came as a blow to them. They said that they developed fear because they did not know what to expect.

*‘… Yes uhmm…. So like the way he has colon cancer we didn’t know we thought he had gases and the way the stomach is paining so by the time he started coming to the hospital he wanted to know what was causing his stomach to pain and we found out it was colon cancer’. Respondent 1*

*‘I wonder if my husband will ever be cured. When I ask the medical personnel they don’t give me satisfying answers’. Respondent 5*

*‘It is a challenge you know... Ummh... I am learning new things everyday’ Respondent 10.*

*‘Ummm...been in the hospital those days I was scared cleaning in the hospital looking after patients buts now I have experience of seeing patients who are critically ill, but now I have adapted to that, those days I was so so scared to stay in the hospital to look after patients’ Respondent 6.*

Some participants also said that the cancer diagnosis disturbed their plans, which they had made, and brought a new understanding to say that this world has many problems, which people go through.

*‘Cancer has also disturbed the plans we had because right now we are supposed to be helping the family working so... Getting some finances and forge ahead now we are here’. Respondent 7*

*‘Uhmm...awe I’m failing to say I have learned a lot...uhmm but on this earth they are a lot of different problems here at the hospital where I came’ Respondent 9.*

### Facilitators/benefits

#### Knowledgeable about cancer and infection prevention

The women reported that they learnt many things during their care of their loved ones; they itemised an increase in knowledge, especially about cancer and infection prevention. They stated that they learnt about cancer, its predisposing factors and treatments.

*‘Most of the times you find that like this we are in hospital uhmmm…. You learn what cancer is, the treatments. The benefits I am finding mostly is learning about cancer, how cancer starts, if you haven’t treated it early … yes uhmm…. So like the way he has colon cancer we didn’t know we thought he had gases and the way the stomach is paining so by the time he started coming to the hospital he wanted to know what was causing his stomach to pain and we found out it was colon cancer’. Respondent 1*

*‘I have learnt a lot about cancer if he wasn’t sick I was not going to know about all this, I have learnt about a lot of things, on the side effects of chemotherapy, like vomiting, headache, diarrhea, also our relationship is strong because am always with him and do everything for him’. Respondent 2*

#### Infection prevention

The women said they learnt how to keep their patients and environment clean, because they interacted with and observed health care staff as they kept the environment clean. Some participants highlighted that they were able to learn how to take care of their patients.

*‘I have learnt a lot about hygiene to take care of where we sleep, how to wash hands, yes that is what I have learnt, also taking care of the patient, the patient has to be clean’. Respondent 3*

*‘Ummm, the place is clean there, the beddings sometimes they come and do the bed the nurses, if you are not around, they do the bed, the making of the bed, those were the benefits’ Respondent 6.*

#### Strong marital relationship

*Reinforced marital emotional bond.* Participants highlighted that taking care of their husbands with cancer strengthened their marital relationships. This was attributed to them always being with their husbands and doing everything for them, since they could not do that for themselves.

*‘Mostly... The benefit is that we have become closer than before with my husband’ Respondent 10.*

*‘I have learnt a lot about cancer ……. Also our relationship is strong because I am always with him and do everything for him’. Respondent 2*

*‘The benefits? eeeh... The benefit is that eeeh... As a wife when you are near your husband you feel comforted, so it is a benefit to me yeah’ Respondent 7.*

Participants highlighted that a cancer diagnosis enabled them to spend more time with their husbands and perform many activities together. This strengthened their marital bond because they would take walks outside together and that was a source of their strength throughout their caregiving role.

*‘Right now I’m no longer found with my friends, I’m just found with my husband at home’ Respondent 14.*

*‘We go for walk with my husband just outside, we also pray, when his not very ill we go outside, not just sleeping in bed, when am not very ill I tell her stories and she feels good ,unlike when am low she feels lonely’ Respondent 3.*

Some participants also mentioned that when they were at home, they were busy with activities other than their relationships.

*‘I have gotten to spend more time with my husband unlike before just home, because at home we are busy with other things, rather than us’. Respondent 4*

#### Development of virtues

*Tolerance and perseverance.* The women highlighted that throughout the care of their loved ones, they learned tolerance and perseverance. They explained that their husbands were easily irritated, angered, stubborn and frustrated because of their condition, and because they were close caregivers, they suffered the consequences of the aforementioned characteristics. It was important to learn that the participants reported that they understood what their husbands were going through and were able to learn to live them.

*‘When he is very angry at the world and everything. I have learned how to be patient with him in all this period. Imh... I am learning new things every day’. Respondent 10*

*‘I’ve learnt a lot because sometimes he can irritated, sometimes stubborn, sometimes wakes up angry.so I just take him like a baby because a baby sometimes can cry his baby’ Respondent 15.*

*‘….. But mostly its anger issues my husband gets to get angry whenever he’s in pain and takes it out on me. I’m learning on how to manage any anger issues and how to handle my husband when experiences pain’. Respondent 10*

#### Resilience and hope

The women reported developing resilience and hope in their situations. Regarding resilience, these participants stated that they had become stronger than before, and they multitasked. Regarding hope, they reported having a positive outlook despite the current circumstances.

*‘My experience has been good, because all I want is for us to find a solution, so that we go home and not something negative’. Respondent 3*

*‘….the challenges are a lot, all I want is for my partner to get well’ Respondent 15.*

*‘We are passing thIgh a lot I tell you... kikikiki (laughing) ……….those are the only challenges, if only you can help us, so that my husband gets well…………..Him he just sleeps. What I want is, help my spouse to get well.’ Respondent 11.*

*‘Mmmmm experiences you really need to be a strong woman because why I say so, they are so many experiences you go through while nursing a patient going through such a condition’. Respondent 14*

#### Good relationships with other caregivers and healthcare workers

The women explained that they experienced a good relationship with health care personnel (HCP). They said that the HCPs were jovial and friendly which made their stay in the hospital interesting. Others stated that even when they wanted to ask something from the HCP, they were not afraid, but they asked and were gladly helped.

*‘The benefits are, you know, you medical personnel are jovial and then you know... even other wives, other bed siders are very helpful you know, when I’m not around when my patient want something like water or he’s on a drip, his able to ask them to give him the water’ Respondent 6.*

*‘The other thing is getting along with your medical personnel, I get along very well with you medical personnel, I get along very well with you when I come there, when I’m asking for something I have never had an experience when am asking a medical personnel Maybe when I need a mop and then they ignore, I have never experienced that’. Respondent 15*

#### I learnt nothing

It is important to report that some women mentioned that they learnt nothing throughout taking care of their husbands with cancer. Others mentioned that the whole caregiving experience had no impact on them because they were able to manage their personal businesses.

*‘I didn’t learn anything’ Respondent 9.*

*‘No, no, there is nothing and benefiting from caring for him’. Respondent 8*

*‘Nothing’ Respondent 3.*

### Coping strategies

Lazarus and Folkman [50] defined coping strategies as purposive cognitive or behavioural changes that enable individuals to deal with external or internal demands. Four themes emerged from what was reported by wives of men with cancer: spiritual support from spiritual carers, prayer and meditation, music and storytelling and social support.

#### Spiritual support from spiritual carers

The women explained that spiritual support from spiritual carers helped them cope by caring for their husbands with cancer. A spiritual carer is a broad term for spiritual leaders in various churches and religious bodies. Some participants also mentioned reading the Bible as a source of their strength.

*‘When we at least talk to our pastor I feel good and better when we tell him what we are going through and when he prays for us I feel better and have hope, yes………yes………’ Respondent 1.*

*‘Because When the church visits me, and share with us the word of God, we get encouraged and feel comforted and strengthened again’. Respondent 7*

*‘I pray, even when we are home, he will be sleeping, I just go to the living room, I do not care whether am alone, I just pray. I switch on the TV and put on Christian channels at least I feel encouraged’. Respondent 2*

#### Prayer and meditation

The women explained that prayer and meditation were sources of their strength. Some stated that when they prayed, they felt much better. Others stated that they would sit alone and meditate, which would strengthen them as they were caring for their husbands.

*‘For sure praying... And that’s the one that gives me energy, strength to overcome that, because when I have prayed I feel at least’ Respondent 7.*

*‘I just pray. I switch on the TV and put on Christian channels at least I feel encouraged’. Respondent 2*

*‘We also pray and when he is not very ill we also go outside to meditate, not just sleeping in bed’ Respondent 3.*

*‘I pray and then I try by all means not to get my husband upset…..ummmm when…. I, I also try to be happy for him’ Respondent 4.*

#### Music and story telling

The women reported that music and storytelling were helpful in helping them cope while caring for their husbands with cancer. Some women said they coped well with gospel music and church songs, while others said they listened to music from the phone without explaining specifically what type of music it was that they were listening to; they all attributed music and storytelling to be the source of their strength in their lives of nursing their husbands with cancer.

*‘Well... I sing gospel music, which helps me cope with the care I give my husband. I also so pray and worship it helps me to cope with the care I give my husband’. Respondent 10*

*‘………..and I sing for my husband church songs as he loves it when I do so and he feels nice’. Respondent 5*

*‘We just sit listen to music from the phone and that helps us cope’ Respondent 12.*

*‘When am not very ill I tell him stories and he feels good, unlike when am low he feels lonely’ Respondent 3.*

#### Social support

The women explained that social support was one of the coping strategies they used, including support from family, friends, church and other social angles. They further stated that social support enabled them to have strength and encouragement in their caring journey.

*‘Ummm the strategies am using...as at now am copying …….with support from family and friends’ Respondent 6.*

*‘Sometimes... Yes, from the family Aaaah... From the church it’s not much’. Respondent 8.*

*‘We get support and encouragement from all angles, Church, friends and staffs here just words It money’. Respondent 3.*

*‘ummmm when…. I, I also try to be happy for him, I also talk to my family and they encourage me’. Respondent 4.*

#### Ignoring and avoiding the distressing situation

Women reported that the coping strategy that they used while caring for their husbands with cancer was ignoring and avoiding the distressing situation. They stated that they just ignored the current situation and also tried to avoid the pain and the anger issues of their husbands, and that enabled them to cope well during the care of their husbands.

*‘I just ignore it, sometimes I would miss a slap sometimes he would bite me just like that I used to cry, cry at home but these days I’m used I just ignore’. Respondent 15*

*‘I try by all means not to get my husband upset…..ummmm when…. I, I also try to be happy for him ………….but you know for his sake and try to stay calm’ Respondent 4.*

*‘But mostly its anger issues my husband gets to get angry whenever he’s in pain and takes it out on me. When he is very angry at the world and everything. I have learned how to be patient with him in all this period’ Respondent 10.*

#### Good marital relationship

The women reported that a good marital relationship helped them to cope despite their husband’s illness. Some participants explained that they walked with their husbands when they felt better and that helped them cope with the care of their husbands.

*‘We go for walk with my husband just outside, when his not very ill we go outside, not just sleeping in bed, when he is not very ill and he feels good, unlike when he is low, he feels lonely, because most of the people here use bemba and he can’t speak so I keep him company’. Respondent 3*

*‘Like’the day when the doctoIaid they are some cancer cells we didn’t sleep that night and... We were not talking to ’ach other just when I woke up I find my husband is awake anytime I’m I woke up I found, find’him his awake not until when we st’rted encouraging each other let’s not give up God is with us if it’s true they will tell us yes and you are going to get treatment yeah’. Respondent 7.*

*‘The experience is okay, because when I look back, he also used to take care of me when he was working so now that his sick, they is nothing I can do but take care of him, am the only one that can do that because his not able to do that on his own and also our relationship is strong because am always with him and do everything for him’. Respondent 2.*

#### Needs of women married to men with cancer

*Financial support.* The women mentioned the financial challenges they faced in their caregiving role. When asked about their needs, most of them mentioned that they had financial needs, meaning that they needed financial support that would enable them to pay for their health care bills, food and household bills whilst in hospital.

*‘I want those at home to help me if they find money. I pay for those 2 months so that I can get my things and take them so that I can continue to look after the patient and see how it will be if we are discharged’ Respondent 15.*

*‘As you see me now I have a lot of needs, I need money to meet our needs in hospital’ Respondent 4.*

*‘Well, we have challenges with transport, ummmm…to move to and from home to the hospital is a challenge at times, finances have become a challenge, ummm…… I need finances and shelter’ Respondent 5.*

*‘M’ need is financial, on finances, ye’, am at the bed side and I don’t have anything to eat obviously it’s all about finances I need to have money’ Respondent 6.*

*‘The other need is finances which we currently are lacking now, it’s hard to buy the food he wants/needs and pay for other hospital expenses also transport and both of us not going for work it’s a challenge…….I also need finances’ Respondent 10.*

*‘Like so the needs I need are many, because we need to take the children to school we have stayed for a long time meaning we won’t be able to take our children to school. Again rentals again food prices have gone up and we do not doing anything so it’s hard’ Respondent 1.*

*Psychosocial counselling.* Many participants expressed the need for psychosocial counselling during the period of nursing their loved ones. They explained that the encouragement that they received from counselling counselling helped them in their caregiving role.

*‘My personal needs? I can say. I need encouragement, well in short yes, I need counselling because you know what uhhh’. Respondent 7.*

*‘when I woke up I find my husband is awake anytime I’m I woke up I found, find him his awake not until when we started encouraging each other, let’s not give up, God is with us. if it’s true you have cancer, they will tell us and you are going to get treatment yes’ Respondent 15.*

*Sexual intimacy and contact.* The women expressed a need for sexual intimacy and contact with their husbands. These participants explained that they were not able to have sexual intercourse with their husbands because of their husband’s cancer status. Others mentioned that the lack of sexual contact affected them greatly.

*‘Like so the needs I have are many……. I need sex also because from the time he got sick I couldn’t have sexual pleasure’. Respondent 1*

*‘As you see me now I have a lot of needs, I need money to meet our needs in hospital, we cannot meet as husband and wife, you know what I mean, because of his condition’. Respondent 4*

*‘Mmmmm….’ I am not able to have sexual intercourse with my husband as at now and it’s a challenge’ Respondent 8.*

*‘Me the needs are to go back for work. I want my husband’s to perform his duties sexually as it is affecting me greatly’. Respondent 10*

*Caregiver accommodation.* The women expressed their need for accommodation where they could sleep due to their husbands’ frequent and sometimes long admissions to the hospital.

*‘You see me here am a visitor, we came from far,…….I need money, where to sleep, I just sleep down’. Respondent 3.*

*‘Well, we have challenges …to move to and from home to the hospital is a challenge at times, finances have become a challenge, ummm…., causing to the fact that we are ever going to the hospital for his treatment. I need shelter’. Respondent 5.*

*Time off from caregiving.* The women mentioned that they needed time off from their caregiving role to do other things aside from caregiving and also to relieve their caregiving burden. These caregivers stated that time off from caregiving would enable them to do other activities to bring food to the table. Also, going time off would enable them to continue their business and enable them to pay household bills and their children’s school fees.

*‘Like so the needs I have are many, because I do business and he also does business so, business it’s like it’s at a standstill, because we need to take the children to school we have stayed for a long time meaning we won’t be able to take our children to school. Again rentals, again food prices have gone up and we are not doing anything so it’s hard…’ Respondent 1.*

*‘Ahhhh... I do not attend church as I used to. It also has a great impact on my occupation as I do not report for work due to my husband’s condition’ Respondent 10.*

*‘The challenges are many..., it’s hard to buy the food he wants/needs and pay ’or other hospital expenses also transport and both of us not going for work it’s a challenge’. Respondent 15*

Apart from participants indicating taking time off to go and do their businesses, they also explained that they needed time off to socialise with their families and friends and attend social gatherings.

*‘I want to go back to my business, it helps out with experiences at home, also you know I want to socialize with my friends and family like I used to before, also I would like to start going to church. I need finances an’ shelter as well’. Respondent 5*

*‘When he is in hospital am always there I can’t go for work am always there by his bed side am not able to go to church….. Am not able to visit my family and friends am not able to move out you know to go out to socialize be with my friends to go to church something like that’. Respondent 6*

*‘My personal needs? I can say hey…I have church needs because when you hear the word of god you get encouraged and feel comforted’. Respondent 7*

*Information need.* The women explained that they had a need to know what their husband was suffering from. They stated openly that they needed to know what their husband’s problem was. Others said, not knowing their husbands’ cancer condition affected them mentally.

*‘I just want to know what the problem is...Mmmm...and...ah...mmm..’. Respondent 9*

*‘I just want to know what the problem is that’s all’. Respondent 15*

*‘This has affected me mentally because I wonder if my husband will ever be cured. When I ask the medical personnel they don’t give me satisfying answers’ Respondent 5.*

*‘When you don’t even know what one is suffering from. Only when you bring someone to the hospital you will know what the problem is’. Respondent 1*

## Discussion

This study explored the experiences and coping strategies of women caring for their husbands with cancer attending CDH in Lusaka, Zambia. The women mentioned multiple challenges during their experiences as caregivers. Their husbands’ frequent hospital admissions were particularly challenging. Similarly, LeSeure and Chongkham-Ang [14] report in their systematic review that caregiver schedules were disrupted because they frequented the hospital where the patients were admitted for treatment. The poor hospital environment the Zambian wives mentioned aligned with the findings of Wang *et al* [25], who stated that Chinese caregivers complained about the hospital facilities and poor health care services they had encountered. It is evident that wives of men with cancer are challenged by their husbands’ frequent admissions, which disrupt their normal family life schedules.

All the women expressed the transportation difficulties they faced whilst caring for their husbands. This finding is similar to Preisler *et al* [26] who reported that caregivers of patients with cancer in Germany suffered many challenges due to illness progression, and their general condition including transportation costs and difficulties because they needed to pay for transportation every time the patients needed to come to the hospital to see their oncologist. Similarly, Tan *et al* [27] reported that mothers of children with cancer in Singapore mentioned many financial problems because of their child’s diagnosis, treatment and transportation and time during the care of their loved ones. Slaboda *et al* [28] agreed that caregivers in the US had difficulties accessing cancer services, which was related to distance, transportation costs and the high cost of health care. Therefore, evidence has emerged that cancer caregivers experience transportation challenges due to financial constraints.

Many participants expressed that they lacked money to meet their needs and their husbands’ needs in our study. This could also be attributed to the fact that the women had left their homes to come and live in the hospital. Others mentioned that the lack of money was a result of frequent hospital admissions, long stays in the hospital and inability to find to find money due to their caregiving role. This is similar to a qualitative study conducted in Indonesia by Sihombing *et al* [29] who stated that caregivers of cancer patients in Indonesia experienced financial burdens and were forced to leave their families, leaving their roles as mothers to accompany their partners as full-time caregivers as they underwent treatment. Wang *et al* [25] also found that Chinese caregivers of patients with cancer experienced severe financial needs because of the demands of a cancer diagnosis and the cancer continuum of care. Therefore, it is evident that caregivers need financial support to effectively care for their loved ones.

Caregivers reported having a serious disruption in their social lives including interactions with family, social gatherings, friends, work, jobs and entertainment. This is similar to Li *et al* [30] who reported that caregivers in Hong Kong experienced significant social disruption due to their caregiving role. Furthermore, in a Singapore study of mothers of children with cancer by Tan *et al* [27] it was reported that the cancer diagnosis of a child with cancer in the family disrupts the social life of caregivers, as they are not able to attend to other social activities, social relationships, or even be able to attend to family relationships and matters, but will use all their energies and time to attend to their sick husband. Slaboda *et al* [28] also reported that caregivers had a significant disruption in their social lives due to their caregiving role. In a qualitative study conducted in Indonesia by Sihombing *et al* [29], they reported that spouses of lung cancer patients specifically women, experiences role changes in addition to what the roles that women naturally possess such as personal care and household activities more than men. Therefore, wives caring for their husbands with cancer in many countries experience disruptions in various aspects of their social lives.

The wives of men in this study with genitourinary and GIT cancers reported having reduced their sexual desire. These findings depart from a findings of the phenomenological study conducted in Ghana by Owoo *et al* [31] about spouses of prostate cancer patients who did not report about reduced sexual desires. LeSeure and Chongkham-Ang [14] agree that the findings of their systematic review indicated that caregivers adapted to the fact that the patients with cancer were not able to adequately perform sexual functions. Correspondingly, Wasner *et al* [32] also reported that caregivers in Germany understood the patients’ situation and were not able to demand sex because they did not have a sexual interest, but would rather remain close to their spouses. Therefore, it is evident that wives of men with some cancers may experience a loss of interest in sexual intercourse because they understand their husbands’ situation.

The women in this study reported that they were burdened when caring for their husbands. This caregiving burden included taking care of their husbands’ needs whilst in the hospital such as bathing and feeding them, finding food for them, offering psychological and emotional support, and finding a place where they themselves could sleep. These findings align with Owoo *et al* [31] findings that spouses of prostate cancer patients in Ghana experience a severe caregiving burden, which leads to a physical impact due to their caregiving role. This is similar to the findings of Cal *et al* [33] who reported that Turkish caregivers complained that their spouses needed care like babies and were overburdened when providing care to their spouses with cancer. They further reported that caregivers complained that they took care of their spouses with cancer as well as managed household chores at the time, which became a burden as well. Wasner *et al* [32] agreed that most caregivers of patients with cancer in Germany felt worse cancer pain when the patients complained than the patients themselves because the patients’ agony became their agony as well. In a secondary analysis of qualitative studies conducted in Nigeria, Uganda and Zimbabwe by Adejoh *et al* [34] it was reported that caregivers took most of the care of their patients with cancer such as medical, physical, financial and emotional needs while sacrificing their employment, finances and their own health and social life. Equally, Ketcher *et al* [35] in the US and LeSeure and Chongkham-Ang [14] in a systematic review agreed that caregivers’ burden disrupted most of their life’s activities and schedules. Therefore, it is evident that wives of patients with cancer experience experience a burdensome caregiving role.

Many women reported having a career halt and stated that everything else stopped, as they focused on caring for their sick husbands. This is similar to Wasner *et al* [32] who reported that many German caregivers reduced their work hours or stopped their various income-generating activities and jobs to concentrate on their patients throughout their illness. Similarly, Tan *et al* [27] report mothers of children with cancer reported having a disruption in their career or employment because of their child’s illness. Slaboda *et al* [28] stated that caregiving has a negative effect on the caregivers’ career in the US because it is demanding of time and energy. The evidence suggests that wives of men with cancer experience career halts and are unable to continue with the various businesses that provide income.

The women in this study stated that they were not able to meet their children’s support due to their caregiving role. This is congruent with Preisler *et al* [26] who reported that German caregivers of patients with cancer experienced psychological pressure that came as a result of the illness of their spouses and also as a result of family characteristics with regard to the role distribution of child care and family care while caring for their spouse with cancer. Studies have implied that caregivers of patients with cancer become so overwhelmed with caregiving they are unable to provide child support. Evidenced has emerged that spouses of men with cancer are unable to provide child support due to the demands of caregiving.

The women in our study explained that caring for their husbands with cancer brought about a new perspective on life. Others said that they initially thought their husbands had problems other than cancer, until the diagnosis was confirmed, which came as a blow to them. Participants said that they developed fear because they did not know what to expect. Some participants also said that the cancer diagnosis disturbed plans they had made, and brought a new understanding that this world has many problems, which people going through cancer treatment go through. Similarly, Cal *et al* [33] reported that a cancer diagnosis brought a whole lot of new changes in the lives of Turkish caregivers and they developed fear because they did not know what to expect during the period when their husbands were going through that. Studies have shown that caregivers reported that they experienced new perspectives and were uncertain about the future [26, 36]. Therefore, it appears that wives of men with cancer experience disruption because of their husbands’ diagnosis and experience.

The participants reported feelings of worry, anxiety, stress, frustration and fear during the care of their husbands. This finding is similar to Sklenarova *et al* [37] who reported that German caregivers of patients with cancer experienced high levels of depression and anxiety symptoms. Wang *et al* [25] agreed that Chinese caregivers reported significantly high levels of psychological distress. Additionally, Preisler *et al* [26] reported that German caregivers experienced similar psychological burdens due to the cancer treatment of their relatives and this psychological burden was expressed as anxiety, hope and helplessness. Nipp *et al* [38] agreed that patient caregivers in the US experienced high levels of depression and anxiety. Many studies report that wives of patients with cancer go through high levels of psychological and emotional distress [35, 38, 39]. Therefore, there is evidence that wives of patients with cancer go through psychological distress, which manifests as worry, stress, anxiety, fear and frustration.

The participants reported needing to know about knowing their spouses’ condition because they had not been involved in decision making. These findings contradicts to a qualitative study by Huang *et al* [40] on spousal caregiving for patients with cancer in a Chinese population who reported that the information of the patients diagnosis was first given to spouses who were responsible for making medical decisions for the patient.

### Facilitators/benefits

The findings of this study highlighted that women taking care of their husbands with cancer strengthened their marital relationships. This was attributed to always being with their husbands and doing everything for them since they could not do many tasks for themselves. The participants highlighted that a strong marital relationship also enabled them to take walks outside together and was a source of their strength throughout their caregiving role. These findings align with a systematic review and metasynthesis of qualitative studies conducted by Zeng *et al* [41] regarding family caregivers’ experiences of caring for head and neck patients, who noted that many spouses reported having marital growth because of enhanced communication. This is similar to a Zimbabwean study of caregivers of children with cancer conducted by Dambi *et al* [36] and USA study of spousal/partner caregivers conducted by Trudeau-Hern and Daneshpour [42] who reported that caregivers experienced a positive impact on their marriage in that their marriage changed for the better following a cancer diagnosis. Similarly, Cal *et al* [33] stated that Turkish caregivers reported that during the care of their husbands with cancer, they experienced good communication in their marriage as they talked more frequently than they had done before and they spent quite a significant amount of time together which made be very close to each other. Equally, LeSeure and Chongkham-Ang [14] reported that across their systematic review, a strong marital relationship developed because of spousal caregiving for the one with cancer. Therefore, it appears that a cancer diagnosis in a marital relationship can strengthen the marital bond between two people.

The findings of this study highlight that the women learned tolerance and perseverance throughout the care of their loved ones. They explained that their husbands were easily irritated, angered, stubborn and frustrated because of their condition, and because they were close caregivers, they suffered the consequences of the aforementioned characteristics. This is similar to a Turkish study by Cal *et al* [33] who stated that caregivers reported doing everything that their spouses requested of them while being patient with them and without showing anger or being overwhelmed. LeSeure and Chongkham-Ang [14] found in their systematic review that many caregivers stated tolerance and perseverance as attributes learnt throughout their caregiving role. It is therefore evident that a cancer diagnosis can teach caregivers of patients with cancer to have perseverance and tolerance.

The women in this study reported developing resilience and hope in their situations. They stated that they had become stronger and could multitask. They also reported having a positive outlook despite the current circumstances. LeSeure and Chongkham-Ang [14] reported that caregivers in their systematic review mentioned resilience and hope as positive attributes they attained from the caregiving process. Similarly, Cal *et al* [33] stated that resilience reported by Turkish caregivers was learnt in their caregiving role. It is evident that resilience and hope can be positive attributes attained throughout the journey of caregiving for patients with cancer.

The women in our study reported that a good marital relationship helped them to cope despite their husbands’ illnesses. Wasner *et al* [32] implied that a concrete relationship with health care professionals (HCP) is required and this comes because of frequent admissions due to a cancer diagnosis. Similarly, Tan *et al* [27] reported that caregivers shared great experiences that they had with HCP throughout their illness. They stated that HCP were very helpful and attended to them whenever they needed help. Cal *et al* [33] also reported that Turkish caregivers were very satisfied with the health care service they had received. It is evident that caregivers often report good relationships with HCP and other caregivers, which was also found in this study.

Regarding hospital cleanliness, some participants complained of being in a poor hospital environment with inadequate water supply, dirty water on the hospital floor, and dirty toilets and bathrooms, which seemed to be a challenge for them. The Zambian women’s distress was similar to patients with a similar experience on an oncology ward in Kenya [43] and hospitals in South Africa [47]. Considering that CDH is a hospital caring for an entire patient population who are immunosuppressed, unclean bathroom facilities are potential sources of life-threatening infections. In 2019, ‘the WHO/UNICEF Joint Monitoring Programme for Water Supply, Sanitation and Hygiene published the Global Baseline Report on WASH in health care facilities, the first ever harmonised estimates for water, sanitation, hand hygiene, health care waste management, and environmental cleaning services in health care facilities across the world’ [7]. A survey, site visits and analysis of the situation in 19 Eastern and Southern African countries found that only three had ‘…O&M [operations and maintenance] plans specifying the roles and responsibilities of actors at national, sub-national, hospital, health facility and health post levels’, which unfortunately, did not include Zambia [7]. The report notes the key role of robust WASH facilities in preventing hospital-acquired infections.

### Coping strategies

The coping strategies that wives of men with cancer used to stay strong throughout their distressing situations included spiritual support. This is similar to a study by Vinci *et al* [44] who reported that caregivers of patients who had received a bone marrow transplant in the US used coping strategies, such as spirituality (care from spiritual carers), to enable them to go through the caregiving period. Tan *et al* [27] also reported that mothers of children with cancer used spirituality as their source of strength to go through their difficult times. LeSeure and Chongkham-Ang [14] mentioned in that the findings of their systematic review indicated that spiritual support for wives of men with cancer helps them to cope with their distressing situation. Therefore, spiritual support is a supportive coping mechanism that helps spouses of men and mothers of children with cancer feel strengthened throughout distressing situations.

The women interviewed in our study explained that prayer and meditation were sources of their strengths. Some stated that when they prayed, they felt much better. Others stated that they would sit alone and meditate, which would strengthen them as they cared for their husbands. This is similar to findings by Vinci *et al* [44] who reported that US caregivers used coping strategies such as spirituality to find strength and help them cope with their hard situations. Similarly, Cal *et al* [33] reported that Turkish caregivers used prayer as their source of strength and this enabled them to go through their caregiving role. Evidence has emerged that prayer and meditation are helpful coping mechanisms for wives of men with cancer.

The women in this study reported that music and storytelling were helpful while trying to cope when caring for their husbands with cancer. Some women said they coped well with gospel music and church songs, while others said they listened to music from the phone without explaining specifically what type of music they were listening to; they all attributed music and storytelling to be the source of their strength in their time of nursing their husbands with cancer. This is similar to Vinci *et al* [44] who reported that US caregivers used coping strategies such as hobbies to find strength and help them cope with their hard situations. Music and storytelling are good coping mechanisms for wives of men with cancer.

The Zambian women in this study explained that social support was one of the coping strategies they used; including support from family, friends, church and other social angles. They further stated that social support enabled them to have strength, hope and encouragement. This is similar to Cal *et al* [33] who stated that Turkish caregivers reported visitors cheered them up and boosted their morale to support their spouses with cancer and family support relieved their distress from caregiving and helped them to smoothly go through their period of caring for their spouses with cancer. Vinci *et al* [44] reported that caregivers in the US used coping strategies such as social support to find strength and help them cope with their hard situations. LeSeure and Chongkham-Ang [14] further add that caregivers across a systematic review reported that social support from friends, family and others gave them strength to go through their distressing situation. The findings of this study differ from findings of a study conducted in South Africa by Maree *et al* [45] who stated that the some primary caregivers used smoking and recreation as a coping mechanism, which may be attributed to the difference in culture between Zambia and South Africa. Social support can act as a good coping mechanism for wives of men with cancer.

Women in this study mentioned that their coping strategy while caring for their husbands with cancer was ignoring and avoiding the distressing situation. They stated that they just ignored the current situation and tried to avoid the pain, and anger issues of their husbands, which enabled them to cope well with their husbands’ care. Similarly, Vinci *et al* [44] reported that US caregivers used coping strategies such as avoidance to cope in their difficult times to find strength and help them cope with their hard situations. Correspondingly, LeSeure and Chongkham-Ang [14] reported that across multiple studies many caregivers stated ignoring and avoiding the distressing situation including discussing cancer as a coping mechanism. It is evident that wives of men with cancer may use ignoring and avoiding distressing situations as a coping mechanism.

The women in this study reported that a good marital relationship helped them cope despite their husbands’ illnesses. This finding is similar to Tan *et al* [27] who reported that some mothers of children with cancer in Singapore who were caregivers found good marital relationships were expressed by good communication and love for one another, which were very helpful and gave them strength to go through their trying period. LeSeure and Chongkham-Ang [14] also showed that a close marital relationship is a great source of strength for women caring for men with cancer. A good marital relationship has been shown to be a good coping mechanism for wives of men with cancer. In a Ugandan study conducted by Kizza and Muliira [46], a home-based education intervention delivered to patients and their caregivers positively influenced the caregivers’ knowledge of pain management while at home, and further suggests that cancer pain management educational interventions should be delivered at home, especially in resource-limited settings like Uganda and Zambia.

### Study strengths and limitations

This study reported the experiences and coping strategies of women caring for their husbands with cancer and thus informs health care workers and institutions on what spouses of men with cancer experience, to serve as a basis for finding strategies for improving nursing and healthcare professional practices. Our findings have added to the body of knowledge that informs the development of strategies and hospital policies regarding the care of caregivers of patients with cancer. Furthermore, findings from this study may be used to make recommendations for hospital guidelines and strategies for caring for caregivers in other resource-limited settings.

Participants were recruited only from the CDH in Lusaka, Zambia; therefore, a few opinions cannot represent many other responses from other people. There could be more than one interpretation of the narratives; however, transferability of qualitative research findings may be possible for other cancer hospitals with similar characteristics.

## Conclusion

The research findings presented here reveal the challenges, facilitators and benefits of the experience of women caring for their husbands with cancer attending the CDH in Lusaka, Zambia, as well as their coping strategies. The challenges of mobility difficulties and hospital admissions/problems, socioeconomic problems, psychological and emotional distress, caregiving burden and spiritual anguish were similar to selected studies from other countries including Europe and Asia. Facilitators of the women’s experiences included knowledge about cancer and infection prevention, a strong marital relationship, tolerance and perseverance, resilience and hope, and good relationships with other caregivers and health care workers. Coping strategies when caring for a husband with cancer included spiritual support from spiritual carers, prayer and meditation, music and storytelling, and social support. For oncology nurses caring for men with cancer who are married, the knowledge of the experiences and coping strategies of their wives will allow them to be more proactive in their nursing care, not only by looking at the patients but also holistically assessing their spouses at the time of the men’s admission to the hospital. The nurses can also routinely assessing the women from time to time, to try to prevent or manage their suffering. Moreover, the women’s coping strategies highlighted and their needs, such as caregiver accommodation in the hospital and spiritual support, need to be availed to spouses caring for men with cancer to smoothen their caring process.

## List of abbreviations

CDH: Cancer Diseases Hospital; GRZ: Government of the Republic of Zambia; GIT: Gastro-intestinal cancers; HCP: Health care personnel.

## Conflicts of interest

The authors declare no conflicts of interest regarding the publication of this paper.

## Funding

No external funding was received for this study.

## Figures and Tables

**Table 1.** Demographic characteristics of participants (*N* = 20).

Variable	Category	Frequency	Percent
Age	20–29	7	35
	30–39	3	15
	40–49	7	35
	50–69	3	15
	Total	20	100

Education	No formal education	2	10
	Primary education	11	55
	Secondary education	5	25
	Tertiary	2	10
	Total	20	100

Religious denomination	Baptist	1	5
	Jehovah’s Witnesses	2	10
	Methodist Church	1	5
	New Apostolic Church	2	10
	Pentecostal	6	30
	Reformed Church	2	10
	Roman Catholic Church	3	15
	Seventh Day Adventist	2	10
	United Church of Zambia	1	5
	Total	20	100

Occupation	Civil servant	3	15
	Self employed	12	60
	Retired		
	Unemployed	5	25
	Total	20	100

Income per month	<K1,000	8	40
	K1,000–K3,000	6	30
	K3,000–K5,000	4	20
	>K5,000	2	10
	Total	20	100

**Table 2.** Cancer and treatment characteristics of husbands of participants.

Variable	Classification	Frequency	Percent
Cancer category	GIT	9	45
	Genital-urinary cancers	4	20
	Skin cancers	2	10
	Haematological cancers	3	15
	Respiratory cancers	0	0
	Head and neck cancer	2	10
	Breast cancer	0	0
	Others	0	20
	Total	20	100

Cancer stage	Stage I	4	20
	Stage II	2	10
	Stage III	4	20
	Stage IV	8	40
	Unknown	2	10
	Total	20	100

Cancer treatment	Chemotherapy	14	70
	Radiotherapy	1	5
	Chemotherapy and radiotherapy	2	10
	Surgery	0	0
	Surgery and radiotherapy	0	0
	Palliative treatment	2	10
	Pretreatment for cancer	1	5
	Total	20	100

**Table 3.** Themes identified from interview data.

Challenges			
Hospital admission	Financial challenges	Transportation	Food
Psychological distress	Child support	Loss of sexual interest	Farming challenges
Caregiver accommodation	Career halt	Health care bills	Poor hospital environment
Manage husband’s side effects	Lifestyle changes	Uncertainties about the condition	Caregiving burden
Spiritual distress			
**Facilitators**			
Increase in cancer knowledge	Strong marital relationship	Knowledge increase in hygiene	Positive outlook (hope) despite the current circumstances
Good relationships with health care workers	Support from others	Good hospital environment	Tolerance and perseverance
Role changes	New perspectives about life occurrences	Nothing	Resilience
**Needs of spouses**			
Inability to continue their businesses	Need to go back for work	Rentals and household expenses	Support for food
Sexual intimacy	Attend social gatherings	Time off from sickness to socialise with friends	Financial support
Overburdened with other things		Need to know about the problem	Psychosocial counselling
**Coping strategies**			
Food	Spiritual support from spiritual carers	Prayer and meditation	Story telling
Good marital relationship	Ignoring	Friendship and family support	Music
**Impact of being a carer on your other roles (such as employment or being a mother)**			
Financial constraint	Everything has stopped	A negative impact on social life	

## References

[1] Aydogan U, Doganer Y, Komurcu S (2016). Coping attitudes of cancer patients and their caregivers and quality of life of caregivers. Indian J Palliat Care.

[2] Cancer Diseases Hospital (2019). Annual Report for 2019.

[3] Milimo MC, Munachonga ML, Mushota L (2004). Zambia Strategic Country Gender Assessment.

[4] Evans A (2014). ‘Women can do what men can do’: the causes and consequences of growing flexibility in gender divisions of labour in Kitwe, Zambia. J South Afr Stud.

[5] Geisler G (1987). Sisters under the skin: women and the women’s league in Zambia. J Mod Afr Stud.

[6] Reigada C, Pais-Ribeiro JL, Novella AS (2015). The caregiver role in palliative care: a systematic review of the literature. Health Care Curr Rev.

[7] Zambia Statistics Agency (2019). 2018 Zambia Demographic Health Survey Summary Report.

[8] Walubita M, Sikateyo B, Zulu JM (2018). Challenges for health care providers, parents and patients who face a child hood cancer diagnosis in Zambia. BMC Health Serv Res.

[9] Sercekus P, Besen DB, Gunusen NP (2014). Experiences of family caregivers of cancer patients receiving chemotherapy. Asian Pac J Cancer Prev.

[10] Tolbert E, Bowie J, Snyder C (2018). A qualitative exploration of the experiences, needs, and roles of caregivers during and after cancer treatment: “That’s what I say. I’m a relative survivor”. J Cancer Surviv.

[11] Takeuchi T, Ichikura K, Amano K (2018). The degree of social difficulties experienced by cancer patients and their spouses. BMC Palliat Care.

[12] Nijboer C, Triemstra M, Tempelaar R (2000). Patterns of caregiver experiences among partners of cancer patients. Gerontologist.

[13] Sibulwa S, Chansa-Kabali T, Hapunda G (2019). “Every part of me has changed” – shared lived experiences of adolescents living with cancer in Zambia. Health Psychol Open.

[14] LeSeure P, Chongkham-Ang S (2015). The experience of caregivers living with cancer patients: a systematic review and meta-synthesis. JPM.

[15] Boatemaa Benson R, Cobbold B, Opoku Boamah E (2020). Challenges, coping strategies, and social support among breast cancer patients in Ghana. Adv Publ Health.

[16] Semenova V, Stadtlander L (2016). Death anxiety, depression, and coping in family caregivers. JSBHS.

[17] Creswell JW (2012). Educational Research: Planning, Conducting, and Evaluating Quantitative and Qualitative Research.

[18] Polit DF, Beck CT (2021). Essentials of Nursing Research: Appraising Evidence for Nursing Practice.

[19] Omona J (2013). Sampling in qualitative research: improving the quality of research outcomes in higher education. Mak J High Educ.

[20] Bryman A (2016). edn (Oxford, New York: Oxford University Press). Social Research Methods.

[21] Walliman N (2010). Research Methods: The Basics.

[22] Forero R, Nahidi S, De Costa J (2018). Application of four-dimension criteria to assess rigour of qualitative research in emergency medicine. BMC Health Serv Res.

[23] Greenhalgh T (2010). How to Read a Paper: The Basics of Evidence-Based Medicine.

[24] Charmaz K (2017). The power of constructivist grounded theory for critical inquiry. Qual Inquiry.

[25] Wang T, Molassiotis A, Tan J-Y (2021). Prevalence and correlates of unmet palliative care needs in dyads of Chinese patients with advanced cancer and their informal caregivers: a cross-sectional survey. Support Care Cancer.

[26] Preisler M, Heuse S, Riemer M (2018). Early integration of palliative cancer care: patients’ and caregivers’ challenges, treatment preferences, and knowledge of illness and treatment throughout the cancer trajectory. Support Care Cancer.

[27] Tan R, Koh S, Wong ME (2020). Caregiver stress, coping strategies, and support needs of mothers caring for their children who are undergoing active cancer treatments. Clin Nurs Res.

[28] Slaboda JC, Nelson SH, Agha Z (2021). A national survey of caregiver’s own experiences and perceptions of U.S. health care system when addressing their health and caring for an older adult. BMC Health Serv Res.

[29] Sihombing Y, Waluyo A, Yona S (2019). The experience of caring for an advanced lung cancer spouse: vulnerable journey of caregiving. Enferm Clín.

[30] Li QP, Mak YW, Loke AY (2013). Spouses’ experience of caregiving for cancer patients: a literature review: stress of spousal caregiving experience. Int Nurs Rev.

[31] Owoo B, Ninnoni JPK, Ampofo EA (2022). “I always find myself very tired and exhausted”: the physical impact of caring; a descriptive phenomenological study of the experiences of prostate cancer caregivers in Cape Coast, Ghana. PLoS One.

[32] Wasner M, Paal P, Borasio GD (2013). Psychosocial care for the caregivers of primary malignant brain tumor patients. J Soc Work End Life Palliat Care.

[33] Cal A, Avci IA, Cavusoglu F (2017). Experiences of caregivers with spouses receiving chemotherapy for colorectal cancer and their expectations from nursing services. Asia Pac J Oncol Nurs.

[34] Adejoh SO, Boele F, Akeju D (2021). The role, impact, and support of informal caregivers in the delivery of palliative care for patients with advanced cancer: a multi-country qualitative study. Palliat Med.

[35] Ketcher D, Trettevik R, Vadaparampil ST (2020). Caring for a spouse with advanced cancer: similarities and differences for male and female caregivers. J Behav Med.

[36] Dambi JM, Makotore FG, Kaseke F (2015). The impact of caregiving a child with cancer: a cross sectional study of experiences of Zimbabwean caregivers. J Palliat Care Med.

[37] Sklenarova H, Krümpelmann A, Haun MW (2015). When do we need to care about the caregiver? Supportive care needs, anxiety, and depression among informal caregivers of patients with cancer and cancer survivors: needs of caregivers of patients with cancer. Cancer.

[38] Nipp RD, El-Jawahri A, Fishbein JN (2016). Factors associated with depression and anxiety symptoms in family caregivers of patients with incurable cancer. Ann Oncol.

[39] Yusuf AJ, Adamu A, Nuhu FT (2011). Caregiver burden among poor caregivers of patients with cancer in an urban African setting: caregiver burden among poor caregivers. Psychooncology.

[40] Huang Y-P, Wang S-Y, Chen S-H (2019). The experience of spousal caregivers of patients recently diagnosed with cancer in Taiwan. Collegian.

[41] Zeng Q, Ling D, Chen W (2022). Family caregivers’ experiences of caring for patients with head and neck cancer: a systematic review and metasynthesis of qualitative studies. Cancer Nurs.

[42] Trudeau-Hern S, Daneshpour M (2012). Cancer’s impact on spousal caregiver health: a qualitative analysis in grounded theory. Contemp Fam Ther.

[43] Mulemi BA (2010). Coping With Cancer and Adversity: Hospital Ethnography in Kenya.

[44] Vinci C, Reblin M, Bulls H (2019). Understanding coping strategies of cancer caregivers to inform mindfulness-based interventions: a qualitative study. Eur J Integr Med.

[45] Maree JE, Moshima D, Ngubeni M (2018). On being a caregiver: the experiences of South African family caregivers caring for cancer patients. Eur J Cancer Care.

[46] Kizza IB, Muliira JK (2019). The influence of a home-based education intervention on family caregivers’ knowledge and self-efficacy for cancer pain management in adult patients within a resource-limited setting. J Cancer Educ.

[47] Nevhutalu HK (2016). ‘Patients’ Rights in South Africa’s Public Health System: Moral-Critical Perspectives’.

[48] Lincoln YS, Guba EG (1985). Naturalistic Inquiry.

[49] Nowell LS, Norris JM, White DE (2017). Thematic analysis: striving to meet the trustworthiness criteria. Int J Qual Methods.

[50] Lazarus R, Folkman S (1984). Stress, Appraisal, and Coping.

